# Neuronal Cell Death and Mouse (*Mus musculus*) Behaviour Induced by Bee Venom

**DOI:** 10.21315/tlsr2018.29.2.1

**Published:** 2018-07-06

**Authors:** Rian Oktiansyah, Berry Juliandi, Kanthi Arum Widayati, Vetnizah Juniantito

**Affiliations:** 1 Department of Biology, Faculty of Mathematics and Natural Sciences, Bogor Agricultural University, Bogor 16680, Indonesia; 2 Department of Clinic, Reproduction and Pathology, Faculty of Veterinary Medicine, Bogor Agricultural University, Bogor 16680, Indonesia

**Keywords:** Cell Death, Mice Behaviour, Bee Venom

## Abstract

Neuronal cell death can occur in a tissue or organ, including the brain, which affects memory. The objectives of this study were to determine the dose of bee venom that causes neuronal death and analyse the alteration of mouse behaviour, focusing in particular on spatial memory. Fifteen male mice of Deutsche Denken Yoken (DDY) strain were divided into control and treatment groups. Bee venom was injected six times for two weeks intraperitoneally with 1.88 mg/kg, 3.76 mg/kg, 5.6 mg/kg, and 7.48 mg/kg doses of venom. Brain histology was studied using haematoxylin-eosin stained paraffin embedded 5 μm coronal sections. A Y maze test was used to assay behaviour. Parameters observed were the number of dead neurons and the percentage of mice with altered behaviour. ANOVA showed that the effects of bee venom were significantly different in the case of the neuronal death parameter but were not significantly different in the case of the mice behaviour parameter. Duncan’s Multiple Range Test (DMRT) demonstrated that P4 (7.48 mg/kg) gave the highest effect of bee venom to promote neuronal death.

## INTRODUCTION

Cell death in multicellular organisms plays an important role during development. Cell death controls the number of cells and protects the organism by removing all cells damaged by disease, ageing, infections, genetic mutations, and exposure to toxic substances. Cell death in nature is categorised into two types: necrosis and apoptosis (Saikumar & Venkatachalam 2009).

Necrosis and apoptosis in response to external factors such as toxic substances and allergens are commonly induced responses (Cudrici *et al.* 2006). Morphologically, necrosis is indicated by shrinkage, fragmentation, or fusing the nucleus of the cells. In contrast to necrosis, apoptosis is characterised by the presence of blebs on the plasma membrane and separation of cytoplasm and organelles of cells (apoptotic body) (Majno & Joris 1995). Necrosis and apoptosis can occur in the same tissue or organ, including in the hippocampus that affects memory.

Memory has an important function for humans, particularly related to intellectual performance and human behaviour. Memory is used for repeating previously performed activities and information retrieval, such as time, directions, purposes, or other important data (Langley 2012; Banikowski 1999). There is a product alleged to affect memory that it is not reported yet scientifically: bee venom.

Bee venom is used as an alternative medicine as well as for the prevention of various diseases, such as arthritis, rheumatism, pain, cancer and neurodegenerative diseases (Kwon *et al.* 2001; Yang *et al.* 2010; Worlitzer *et al.* 2012; Bellik 2015). Bee venom contains a complex mixture of compounds, including polypeptides, enzymes, lipids, and amino acids. Some of these contents provide anti-inflammatory and anti-cell damage effects (Ovcharov *et al.* 1976; Ownby *et al.* 1997; Truskinovsky *et al.* 2001; Raghuraman & Chattopadhyay 2006; Ali 2012; Abdu & Alahmari 2013; Bogdanov 2015; Eze *et al.* 2016; Lee & Bae 2016). Bee venom is produced by two glands in a worker bees sting. Bee venom production increases during the first week of an adult bee worker life and reaches a maximum amount when the worker bee is involved in nest defence and forage. However, bee venom production decreases with age (Krell 1996). There are several studies demonstrating that bee venom can cause cell death.

Bee venom induces cell death in human lymphocytes (Gajski & Garaj-Vrhovac 2011). Bee venom also contains Phospholipase A, which has been shown to induce cell death by inactivating p38 MAPK (Jeong *et al.* 2011). Associated with bee venom’s mode of action as an allergen, there are few studies on allergens resulting in cell death, such as NFAT (Nuclear Factor of Activated T Cell), which triggers inflammation in the skin and can cause cell death (Kwon *et al.* 2016). Mice were injected with 2.4-dinitrofluorobenzen, which induces keratinocyte apoptosis due to the expression of First Apoptosis Signal (FAS) (Hedrych-Ozimina et al. 2011). Nevertheless, bee venom has not been reported yet scientifically to cause neuronal cell death, affecting the behaviour of mice. The objectives of this study are to determine the dose of bee venom that causes neuronal cell death and analyse the alteration of the behaviour of mice, particularly spatial memory.

## MATERIALS AND METHOD

### Time and Place

The study was conducted at the Division of Biological Function and Animal Behaviour, Biology Department, Faculty of Mathematics and Natural Sciences and Pathology Laboratory, Faculty of Veterinary Medicine, Bogor Agricultural University. The study conducted from October 2015 until March 2016.

### Research Animal

Mice used in this study were three-month old male Deutsche Denken Yoken (DDY) mice, approximately 20–30 g in weight. Mice were obtained from the Non-Ruminant Laboratory and Animals Prospect, Faculty of Animal Sciences, Bogor Agricultural University.

### Experimental Unit

Fifteen mice used in this study were divided into control and treatments groups. Each treatment group consisted of three mice. Mice were reared in different cages, according to the group.

### The Treatments of Animals

This study was approved by the ethics committee of Bogor Agricultural University (No. 6-2016 RSHP FKH-IPB). Mice were acclimatised for a week before the treatment. Mice were maintained in the laboratory at room temperature with wood chips. Mice were given water ad libitum throughout the study (Clark *et al.* 2008). Bee venom was injected intraperitoneally after acclimatisation. Bee venom was injected for two weeks (days 1, 4, 7, 10, 13, and 16) with the following doses: 1.88 mg/kg, 3.76 mg/kg, 5.6 mg/kg, and 7.48 mg/kg, respectively (Bogdanov 2015). The bee venom doses used in this study were varied from a minimal dose, an optimal dose, and an exceeds optimal dose to allow study of its effect on cell death. The highest dose selected was considered high enough to cause cell death. It was possible to see a dose dependency of neuronal cell death at higher concentrations. However, the doses administered were sufficient to observe the effect of bee venom on neuronal cell death. Bee venom was obtained from an apitherapy clinic. Morphological examination of brain tissue was performed using sectioned tissue, stained with haematoxylin-eosin (HE). Behaviour test was performed after injection. The purpose of the behaviour test is to examine the spatial memory of mice using a Y-maze (Onaolapo *et al.* 2012). Behaviour tests were performed between 1.30 to 4.30 pm (Juliandi *et al.* 2015). This study is designed as preliminary screening study. This research is expected to provide early information allowing further research using additional behaviour tests and to determine optimal doses of bee venom. Brain tissue isolation was performed three days after the last injection of bee venom using the perfusion method (Matsuda 2007; Erwin *et al.* 2013).

### Experimental Design and Data Analysis

This study used a completely randomised design method that consisted of five treatments and three repetitions. The parameters observed were the percentage of mice to explore the Y maze correctly and the number of dead neurons. Data were analysed using analyses of variance (ANOVA).

## RESULTS

### Mice Brain (Dentate Gyrus) Histology

**Figure 1:**
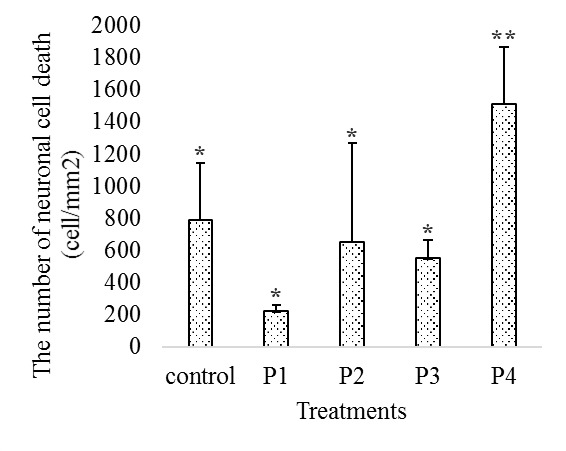
Mean of neuronal cells death in dentate gyrus after 2 weeks of bee venom injections with the following doses P1 = 1.88 mg/kg, P2 = 3.76 mg/kg, P3 = 5.6 mg/kg, and P4 = 7.48 mg/kg

**Figure 2:**
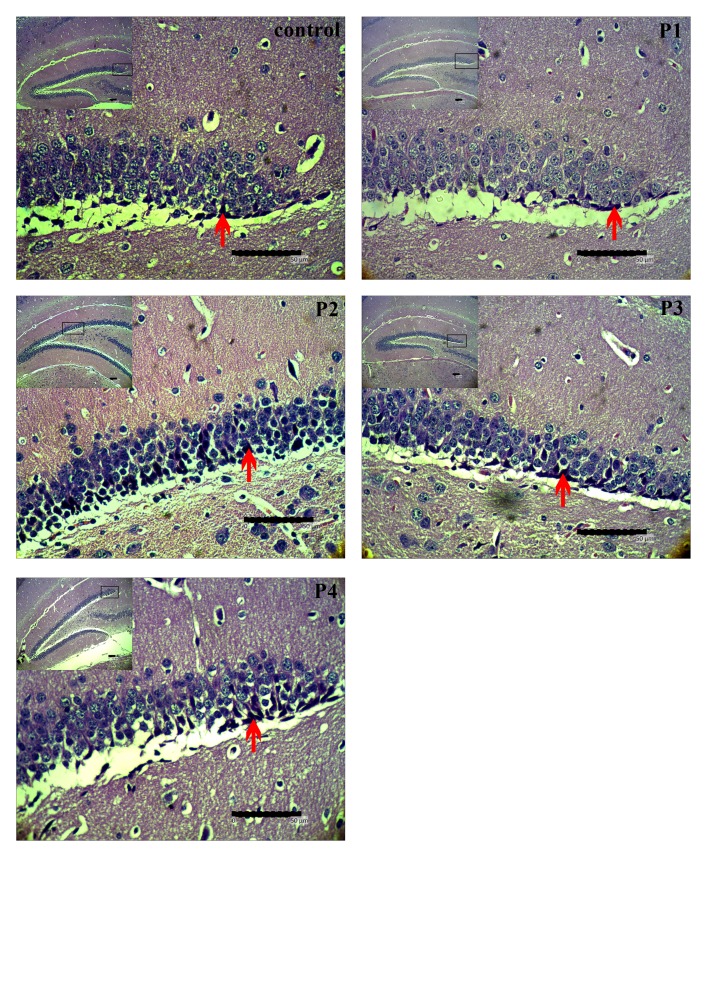
Representative image of brain sections containing dentate gyrus in each treatment stained by HE. Bee venom doses P1 = 1.88 mg/kg, P2= 3.76 mg/kg, P3=5.6 mg/kg, and P4=7.48 mg/kg. Dead neurons are shown by arrow (bars: 50 μm)

Based on observation on histological sections, there was neuronal cell death in the dentate gyrus (Fig. 2). The results showed differences in the average number of dead neuronal cells in each dentate gyrus section. The average number of dead neuronal cells fluctuated in each treatment compared to control. The highest average of dead neurons was 1510 cell/mm^2^, and the lowest average was 220 cell/mm^2^. The fluctuation of the average number of dead cells demonstrated that bee venom can lead to neuronal cell death in the dentate gyrus. The fluctuation of these averages can be seen in Fig. 1.

Fig. 2 demonstrated that neuronal cells death in the dentate gyrus. Dead neurons were marked by cells with dark staining and the absence of a nucleus. ANOVA showed that the effect of bee venom lead to a significantly different number of dead neuronal cells in the dentate gyrus. This suggests that bee venom has a role in the regulation of neuronal cell death in the dentate gyrus. Thus, the Duncan’s Multiple Range Test (DMRT) was required to further examine the effect of treatments on the regulation neuronal cell death in the dentate gyrus.

The DMRT results demonstrated that P4 was significantly different from other treatments. Treatment with this highest dose of bee venom led to the highest number of neuronal cell deaths in the dentate gyrus. Based on this study, many doses of bee venom gave the significant effect on neuronal cells death. Furthermore, the effect of bee venom on neuronal cell death was significant using P4 (7.48 mg/kg).

### Mice Behaviour

**Figure 3:**
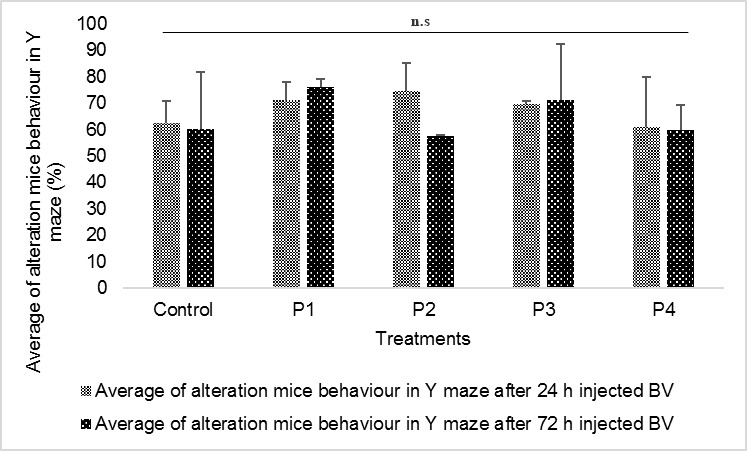
Mean of correct alteration in Y-maze arms. Bee venom doses P1 = 1.88 mg/kg, P2 = 3.76 mg/kg, P3 = 5.6 mg/kg, and P4 = 7.48 mg/kg.

The movement of mice through the Y-maze arm was tracked in this study. The results displayed differences in the average percentage of alteration in mice behaviour. The percentage of alteration in mice behaviour in the Y-maze fluctuated in each treatment compared to control. The highest average of alteration after 24 h bee venom injection was 74.57%, and the lowest was 61.07%. The highest average of alteration after 72 h bee venom injection was 76.03%, and the lowest was 57.50%. The fluctuation of the average showed that bee venom has an influence on altering the behaviour of mice in the Y-maze. This fluctuation can be seen in Fig. 3.

Analysis of variance (ANOVA) showed that the effect of bee venom was not significantly different between mice treatments. Thus, bee venom has no influence on mice behaviour. Although bee venom did not significantly alter mice behaviour, the percentage of alteration mice behaviour in the Y-maze, in addition to the number of neuronal cell deaths in the dentate gyrus indicates that bee venom has an effect.

## DISCUSSION

### Neuronal Cell Death in the Dentate Gyrus

Based on this study, bee venom affects neuronal cell death in the dentate gyrus. The DMRT test showed that the highest dose of bee venom was significantly different from other treatments on neuronal cell death in the dentate gyrus. A dose of 4 mg/kg body weight in rats is the maximum dose of bee venom required to stimulate an anti-inflammatory effect, while the higher dose shows neurotoxic effects (Bogdanov 2015). Fig. 2 demonstrates that neuronal cell deaths can be found in the subgranular zone (SGZ). According to the study, we proposed that the dead neurons are neural progenitor cells (NPC) because they were found in the innermost layer of SGZ. However, special markers would be needed to confirm these cell types.

The increase in the number of neuronal cell deaths in the dentate gyrus after P4 treatment suggested that there may be an influence of inflammatory compounds in the bee venom. Previous studies have demonstrated the role of melittin, phospholipase A2, apamin, and adolapin in high doses contained in bee venom to cause allergies, lysis of erythrocytes, myonecrosis, and other neurotoxic effects (Ovcharov *et al.* 1976; Ownby *et al.* 1997; Raghuraman & Chattopadhyay 2006; Ali 2012; Abdu & Alahmari 2013; Elhakim *et al.* 2014; Bogdanov 2015; Eze *et al.* 2016; Lee & Bae 2016). Therefore, bee venom can affect the peripheral tissues.

The influence of bee venom in peripheral tissues could be expected to produce cytokines that signal the brain, causing neuronal cell death in the dentate gyrus. Dantzer *et al.* (2008), Dilger and Johnson (2008), and Maier and Linda (2012) demonstrated that cytokines from peripheral tissues transported to the brain via the endocrine system can induce neuronal cell death. Palombella and Vilcek (1989) reported that melittin and phospholipase A2 can activate cytokines, in particular TNF. Although this still needs further confirmation, the administration of bee venom is also supposed to indirectly effect neuronal cell death in the dentate gyrus via cytokine signalling.

Another suggestion about the mechanism of neuronal cell death induced to bee venom, is the ability of bee venom compounds to pass through the blood brain barrier (BBB). The BBB plays an important role in regulating the molecules that can pass into and out of the brain (Ransohoff & Engelhardt 2012; Takeshita & Ransohoff 2012). Oller-Salvia *et al.* (2013) and Mourre *et al.* (1997) reported that apamin, a compound of bee venom, can pass through the blood brain barrier and cause neuronal cell death. Other compounds of bee venom, however, have not been reported yet to pass through the BBB. Therefore, apamin could be the causative agent of neuronal cell death in the dentate gyrus.

There was no linear relationship on neuronal cell death in the dentate gyrus with an increasing dose of bee venom. The higher doses of bee venom did not lead higher numbers of neuronal cell deaths in the dentate gyrus. In this study, P2 showed a higher number of dead neuronal cells compared to control, P1, and P3. This anomalous result of P2 does not affect the conclusions from this study. This may be due to the efficiency of bee venom utilisation in P1 treatments. P1 had the lowest amount of neuronal cell death in the dentate gyrus compared to the other treatments. These results provide straightforward information about the efficiency of administration for the apitherapy clinic and the people who want to utilise bee sting therapy.

### The Spatial Learning Ability of Mice

The spatial learning ability of mice is shown by the correct-arm alternation in Y-maze. The Y-maze was first used to examine the spatial learning underlying mice exploring a new environment. Wolfe (1969) reported that mice prefer to investigate new objects and environments compared with known objects and environments. This study showed that bee venom administration tended to effect the spatial learning ability of mice (Fig. 3). The tendency of these effects can be caused by neuronal cell death in the dentate gyrus. The dentate gyrus plays a role in memory formation, in particular related to cognitive function and spatial memory (Silva *et al*. 1998; Eichenbaum *et al.* 1999). Our results demonstrated that the treatment that induced the most dead neurons had the lowest correct-arm alternation in the Y-maze (Fig. 3). We hypothesize that NPCs made up the bulk of dead neurons in the SGZ. Dead or damaged NPCs will cause mature neurons to be reduced in size while the remaining mature neurons will undergo programmed cell death. This will affect the process of learning and spatial memory in mice.

The ANOVA test showed that the number of neuronal cell deaths was not significantly different with respect to altered mice behaviour, particularly spatial memory. The number of neuronal cell deaths in the dentate gyrus induced by bee venom was still under the threshold level, so it could not affect mice behaviour yet. This is consistent with Conrad and Roy (1993), who reported that as much as 80% of neuronal cell death in the dentate gyrus does not affect spatial memory. Although the ANOVA test showed that there was no significant difference, there was a downward trend in the percentage of correct-arm alternation in the Y-maze.

## CONCLUSION

Based on the study, bee venom has neurotoxic effect because it caused neuronal cell death in the dentate gyrus and behaviour alteration in mice. The dose of bee venom that caused the highest neuronal cell deaths in the dentate gyrus was P4 (7.48 mg/kg). The percentage of mice with altered behaviour in a Y-maze test along with the number of neuronal cells death in the dentate gyrus corroborates this.
